# Fibulin-4 deposition requires EMILIN-1 in the extracellular matrix of osteoblasts

**DOI:** 10.1038/s41598-017-05835-7

**Published:** 2017-07-17

**Authors:** Alvise Schiavinato, Douglas R. Keene, Thomas Imhof, Roberto Doliana, Takako Sasaki, Gerhard Sengle

**Affiliations:** 1 Center for Biochemistry, Medical Faculty, University of Cologne, Cologne, Germany; 2 Shriners Hospital for Children, Portland, Oregon USA; 3 Institute for Dental Research and Oral Musculoskeletal Biology, Medical Faculty, University of Cologne, 50931 Cologne, Germany; 4 Experimental Oncology 2, CRO, IRCCS, National Cancer Institute, Aviano, PN Italy; 5 Department of Biochemistry II, Faculty of Medicine, Oita University, Oita, 879-5593 Japan; 6 Center for Molecular Medicine Cologne (CMMC), University of Cologne, Cologne, Germany

## Abstract

Tissue microenvironments formed by extracellular matrix networks play an important role in regulating tissue structure and function. Extracellular microfibrillar networks composed of fibrillins and their associated ligands such as LTBPs, fibulins, and EMILINs are of particular interest in this regard since they provide a specialized cellular microenvironment guiding proper morphology and functional behavior of specialized cell types. To understand how cellular microenvironments composed of intricate microfibrillar networks influence cell fate decisions in a contextual manner, more information about the spatiotemporal localization, deposition, and function of their components is required. By employing confocal immunofluorescence and electron microscopy we investigated the localization and extracellular matrix deposition of EMILIN-1 and -2 in tissues of the skeletal system such as cartilage and bone as well as in *in vitro* cultures of osteoblasts. We found that upon RNAi mediated depletion of EMILIN-1 in primary calvarial osteoblasts and MC3T3-E1 cells only fibulin-4 matrix deposition was lost while other fibulin family members or LTBPs remained unaffected. Immunoprecipitation and ELISA-style binding assays confirmed a direct interaction between EMILIN-1 and fibulin-4. Our data suggest a new function for EMILIN-1 which implies the guidance of linear fibulin-4 matrix deposition and thereby fibulin-4 fiber formation.

## Introduction

EMILINs (Elastin-Microfibril-Interface-Located-proteINs) comprise a family of three structurally homologous extracellular glycoproteins that were reported to serve as versatile regulators of key cellular events, such as cell adhesion, migration, and proliferation^1–3^, but also as unique modulators of extracellular signaling pathways^4^. EMILINs were found to influence pro-TGF-β processing^5, 6^, modulate Wnt and Hedgehog signaling^7, 8^, activate death receptor mediated apoptosis^9^, and communicate with cells via α4β1 and α9β1 integrin mediated signalling^3^. Despite these unique functions in the regulation of cell fate, little is known about a potential role of EMILINs in the organization of the extracellular matrix (ECM) and therefore their contribution to specialized extracellular supramolecular networks in different tissues. This information is critical for a better understanding of how tissue microenvironments composed of intricate ECM networks control cell fate via cell-ECM communication.

EMILINs are especially characterized by a small cysteine-rich N-terminal module of around 75 amino acids, the EMI-domain^4^. Originally, EMILIN-1 was described as a 115 kDa glycoprotein extractable from chicken aorta only under harsh conditions such as 6 M guanidine HCl containing dithioerythritol^10^. Subsequent studies revealed that this extracellular matrix (ECM) glycoprotein is found in various tissues in association with elastic fibers, where it preferentially localizes at the interface between the fibrillin microfibrillar scaffold and the elastin core^11^. Subsequently, two other ECM proteins with high similarity to EMILIN-1 were identified and therefore named EMILIN-2 and EMILIN-3^12, 13^.

Most insight into the functional role of EMILINs was gained by the generation of knockout mice. EMILIN-1 null mice display mild defects of elastic fibers^14^, increased blood pressure^5, 15^, increased epidermal cell proliferation^3^ and defects of lymphatic vessels^16^. EMILIN-2 function has been mainly investigated in the context of tumor growth and neo-angiogenesis^17^. An EMILIN-2 knockout mouse was recently generated and defects in platelets function and clot formation were reported^18^. Morpholino mediated knockdown of EMILIN-3 suggested a function of this protein during notochord development in zebrafish^7^. EMILIN-3 knockout mice have been established recently, however, analysis of their skin revealed no obvious phenotype, while functional consequences for skeletal tissues were not studied^19^.

We recently demonstrated that both EMILIN-1 and EMILIN-2 are targeted to fibrillin microfibrils in the skin and that fibrillin-1 is required for the proper deposition of EMILINs within the extracellular space^20^. This indicates that EMILIN-1 and -2 alterations may play a role in the pathomechanisms of the fibrillinopathies, where microfibril destabilization due to fibrillin-1 or -2 deficiency leads to multisystemic features characterized by reduced tissue integrity and global activation of growth factor signaling^21^. Recently, the first disease causing mutation in the *EMILIN1* gene was reported in a patient with similar features to Marfan syndrome (MFS) such as aortic aneurysms, skeletal abnormalities and increased skin elasticity which is triggered by fibrillin-1 deficiency^22^. This observed clinical overlap of human EMILIN-1 and fibrillin-1 deficiency underpins our previous findings that EMILIN-1 is targeted to supramolecular networks composed of fibrillin microfibrils and elastic fibers to which it confers functionality.

However, the functional role of EMILINs in the context of the microfibril/elastic fiber system remains largely unknown. In particular the impact of EMILIN deficiency on the skeletal system remains obscure. Currently, there is only sparse information available about the spatiotemporal localization and deposition of EMILINs in the skeletal system. Proteomic analysis revealed that EMILIN-1 is abundant in postnatal mouse cartilage^23, 24^ while EMILIN-3 was originally discovered in human mesenchymal stem cells which were incubated with osteogenic supplements and in a variety of osteoblastic cell lines^13^.

In this work, we wanted to investigate the localization of EMILINs in cartilage and bone and to determine which molecular interactions facilitate their ECM incorporation in this system. Moreover, exploring the role of EMILINs for the normal organization of the ECM produced by osteoblasts, we identified fibulin-4 as a new interaction partner for EMILIN-1.

## Results

### Localization of EMILINs in bone and cartilage

**Figure 1:**
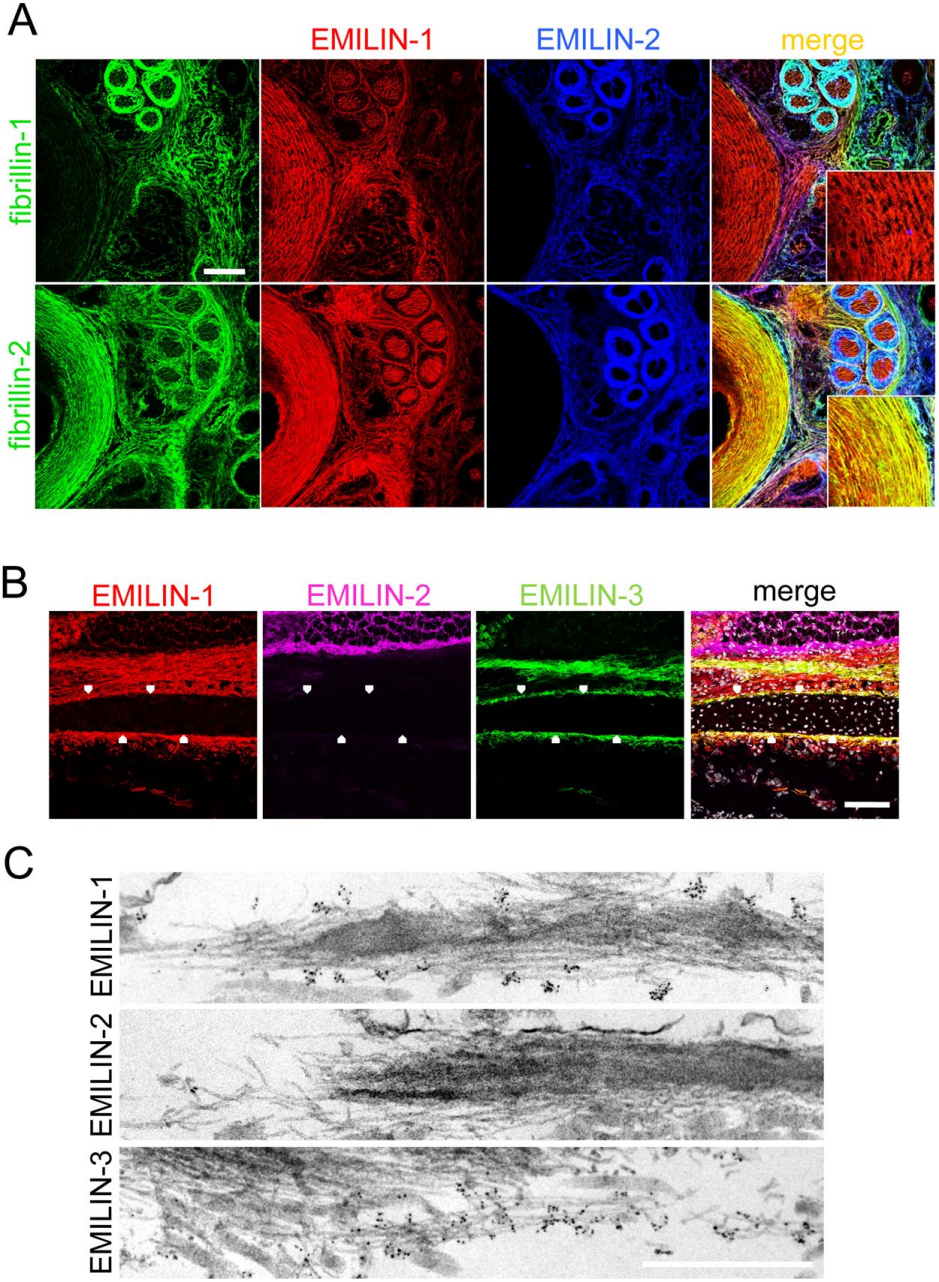
Localization of EMILINs in cartilage and bone. (**A**) Confocal immunofluorescence microscopy showing EMILIN-1 and -2, together with fibrillin-1, and -2 localization in transverse sections from newborn mouse tail. Each protein showed a specific distribution pattern among the different tissues present in the tail. However, signals detected for EMILIN-1 and -2 and fibrillin fibers were consistently overlapping. The insets in the merged panels show a higher magnification of the annulus fibrosus, where EMILIN-1 and fibrillin-2 fibers show co-localization. Scale bar: 75 μm. (**B**) Sagittal section of newborn mouse skull, showing the localization of the three EMILIN proteins by immunofluorescence in calvarial bone. Note the presence of EMILIN-1 and EMILIN-3, but not EMILIN-2, positive fibers in the ECM that directly surround the bone (arrowheads). Scale bar, 75 μm. (**C**) Transmission electron microscopy analysis of newborn mouse trachea, following immunogold labeling for the three EMILIN proteins (black dots). EMILIN-1 and EMILIN-3, but not EMILIN-2, are abundantly distributed on microfibrils. Scale bar, 500 nm.

Our previous studies in murine dermis revealed that the ECM deposition of EMILIN-1 and -2 crucially depends on the successful assembly of fibrillin microfibrils^20^. Therefore we were curious to investigate whether this interdependence also exists in other tissues. Immunofluorescence of transverse sections of newborn mouse tail revealed that the co-localization of EMILINs with fibrillins showed remarkable differences in other tissues than skin (Fig. 1A, Supplementary Fig. S1). For instance, in the fibrocartilage of the annulus fibrosus of the intervertebral disc we found that EMILIN-1 co-localized with fibrillin-2 rather than with fibrillin-1. However, EMILIN-2 showed strong co-localization with fibrillin-1 in the surrounding sheets of tendons (Fig. 1A). These findings prompted us to further investigate the localization of EMILINs in cartilage and bone.

On newborn mouse cryosections we found that EMILIN-1 and EMILIN-3, but not EMILIN-2, are abundantly deposited in the fibrillar material that surrounds calvarial bones (Fig. 1B). A similar distribution pattern was found in the perichondrium and periosteum of different long bones (data not shown). Immunoelectron microscopy confirmed that EMILIN-1 and -3 but not EMILIN-2 are targeted to fibrillin microfibrils in perichondrium of mouse trachea (Fig. 1C).

### Specific role of fibrillins and fibronectin for EMILIN-1 and EMILIN-2 incorporation in the ECM produced by primary calvarial osteoblasts

To gain a better understanding about the role of EMILINs in the formation of bone matrix we studied the *in vitro* ECM deposition of primary calvarial osteoblasts and MC3T3-E1 cells, a pre-osteoblast cell line originally derived from the calvarial bones of newborn mice^25^. Immunofluorescence analysis showed that MC3T3-E1 cells organize an abundant fibrillar extracellular network of EMILIN-1 and EMILIN-2, but do not deposit any EMILIN-3 protein or mRNA even after 10 days of culture (Supplementary Fig. S2A,B). In accordance, we found that primary calvarial osteoblasts assembled EMILIN-1 and -2 fiber networks (Supplementary Fig. S3) but EMILIN-3 fiber formation was negative (data not shown).

**Figure 2:**
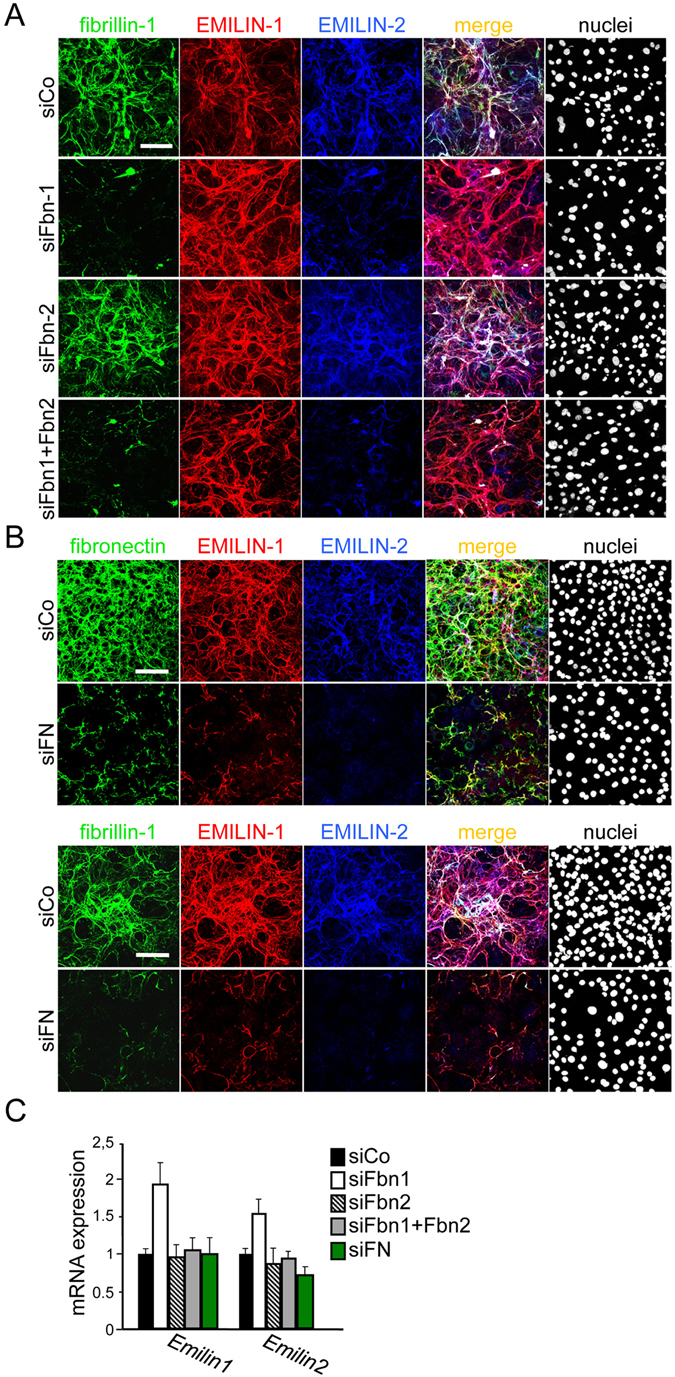
Specific role of fibrillins and fibronectin for EMILIN-1 and EMILIN-2 incorporation in the ECM produced by primary calvarial osteoblasts. (**A**) Confocal immunofluorescence microscopy analysis of *in vitro* matrix fiber formation. Primary calvarial osteoblasts derived from wild type newborn mice were reverse transfected with the indicated fibrillin siRNAs, grown for four days and probed by immunostaining with the indicated antibodies. Knockdown of fibrillin-1 alone was sufficient to impair EMILIN-2 incorporation in the ECM, while even the double knockdown of fibrillin-1 and fibrillin-2 was ineffective in preventing EMILIN-1 matrix deposition. For the double knockdown of fibrillins, a single siRNA for each gene was used. (**B**) siRNA mediated knockdown of fibronectin in primary calvarial osteoblasts was sufficient to completely prevent EMILIN-1 and EMILIN-2, as well as fibrillin-1 fiber formation. (**C**) qPCR analysis on transfected cells revealed that mRNA levels were mostly not affected by siRNA treatment, apart from a slight increase in *Emilin1* and *Emilin2* expression after fibrillin-1 knockdown. All siRNA knock-down experiments were performed three times (n = 3). Scale bars: 75 μm.

We next asked whether EMILIN matrix deposition depends on fibrillin fiber assembly as previously demonstrated for cultured dermal fibroblasts^20^. Similar to our findings with dermal primary fibroblasts^20^, knockdown of one EMILIN in primary calvarial osteoblasts was not affecting fibrillogenesis of the other EMILIN and knockdown of both EMILINs was not affecting fibrillin-1 or fibrillin-2 deposition in primary calvarial osteoblasts or MC3T3-E1 cells (Supplementary Fig. S3, Supplementary Fig. S4). Next we performed siRNA treatments to deplete fibrillins from these cultures to investigate the effect on EMILIN-1 and EMILIN-2 deposition and mRNA levels four days after the reverse transfection. Surprisingly, and differently from what we observed in dermal fibroblasts, even after the depletion of both fibrillins, EMILIN-1 was still incorporated in the ECM produced by primary osteoblasts. On the other hand, depletion of fibrillin-1 was sufficient to completely prevent EMILIN-2 fibrillogenesis while fibrillin-2 knockdown alone had no effect (Fig. 2A, Supplementary Fig. S5). This suggests that extracellular networks assembled by osteoblasts and dermal fibroblasts show significantly different structural characteristics and composition reflecting an adaptation to specialized functions of the specific tissue microenvironments they give rise to.

To test whether fibronectin is required for EMILIN fiber formation we performed fibronectin siRNA mediated knockdown in primary osteoblasts. Interestingly, this treatment was sufficient to impair EMILIN-1, as well as fibrillin-1 and EMILIN-2 assembly (Fig. 2B). Quantitative PCR analysis revealed no major differences in EMILIN mRNAs expression, apart from an upregulation of *Emilin1* transcript in the fibrillin-1 depleted cells (Fig. 2C).

A similar dependence on fibronectin for EMILIN-1 assembly was observed in the ECM of E14 mouse embryonic fibroblasts (MEFs)^20^, suggesting that the architecture of the ECM produced by osteoblasts shows similarities to the ECM during embryonic development. However, only simultaneous depletion of both fibrillins in MEFs clearly affected EMILIN-2 matrix deposition^20^. To gain further insight into the molecular requirements for EMILIN incorporation into the ECM produced by osteoblasts versus fibroblasts we turned to NIH/3T3 embryonic fibroblasts in which we detected no fibrillin-2 fiber assembly (Supplementary Fig. S6A) ruling out any influence of fibrillin-2 on targeting EMILINs in this system. In NIH/3T3 cells, we found that siRNA mediated fibrillin-1 depletion, or heparin addition which was previously shown to inhibit fibrillin-1 fiber assembly^26^ had no effect on EMILIN-1 or EMILIN-2 fiber assembly (Supplementary Fig. S6B,D), however, fibronectin depletion prevented ECM incorporation of EMILIN-1 and EMILIN-2 (Supplementary Fig. S6F). Therefore we conclude that in primary mouse osteoblast cultures, EMILIN-2 incorporation is dependent on fibrillin-1 but not fibrillin-2, while EMILIN-1 is fibrillin independent and requires fibronectin for its assembly. This dependence of EMILIN deposition on fibronectin assembly is in contrast to dermal fibroblasts which require both fibrillins^20^, but similar to embryonic fibroblasts such as NIH/3T3 cells reflecting cell type-specific characteristics in ECM architecture.

### EMILIN-1, but not EMILIN-2, controls fibulin-4 fibril formation in primary calvarial osteoblasts and MC3T3-E1 cells

**Figure 3:**
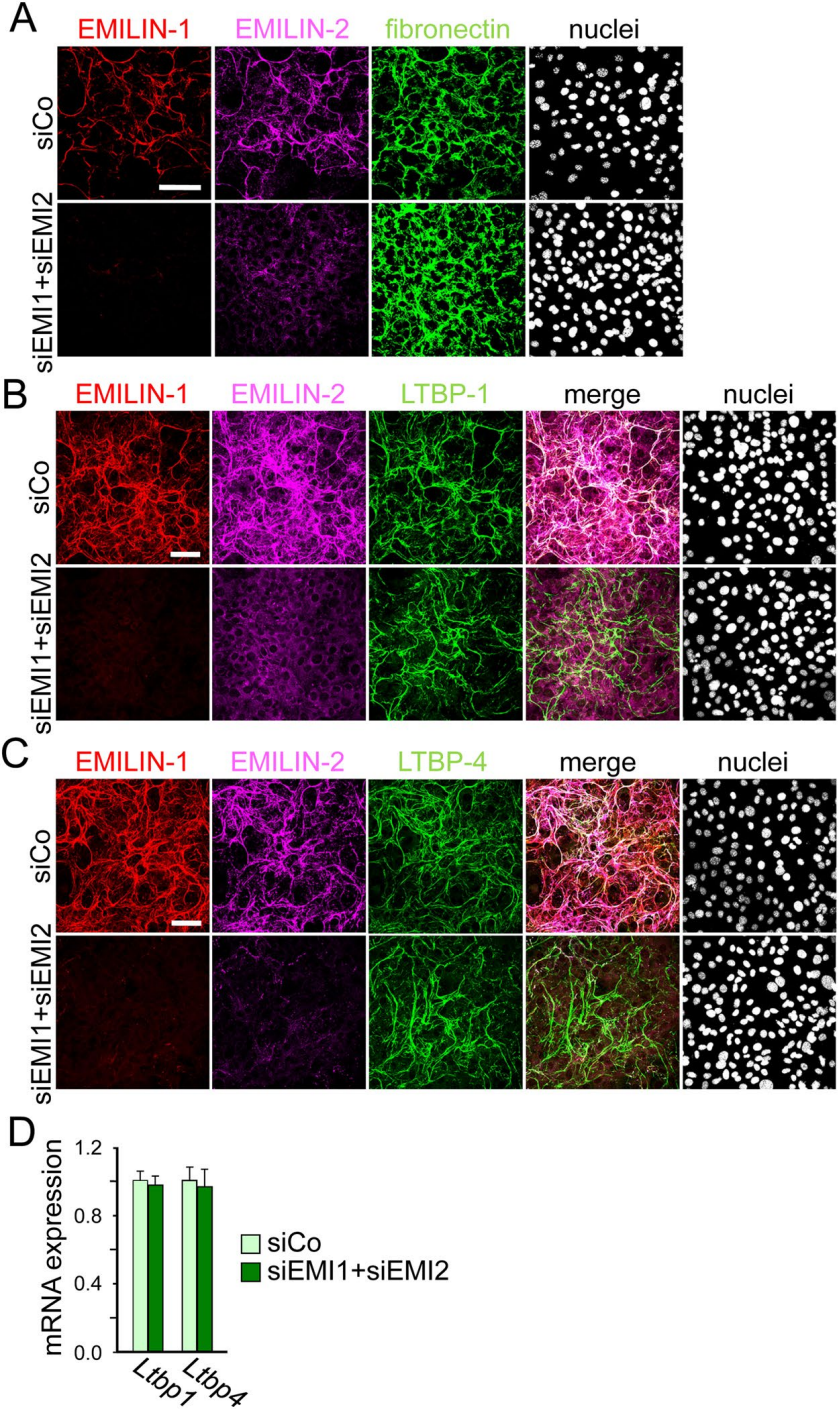
Deposition of fibronectin, LTBP-1 and LTBP-4 in MC3T3-E1 cells after double knockdown of EMILIN-1 and EMILIN-2. MC3T3-E1 osteoblasts were transfected with a mixture of siRNAs against EMILIN-1 (siEMI1) and EMILIN-2 (siEMI2) or with a control siRNA (siCo) and grown for 4 days. (**A**,**B**,**C**) Confocal microscopy of cells transfected with the different siRNAs and immunolabeled with antibodies against (**A**) fibronectin, (**B**) LTBP-1 or (**C**) LTBP-4, Scale bar, 75 μm. Nuclei were stained with Hoechst. (**D**) qRT-PCR analysis of LTBP-1 and LTBP-4 transcripts in cells transfected with the different siRNAs showed no significant differences (n = 3). All siRNA knockdown experiments were performed three times (n = 3).

Next, we investigated whether depletion of EMILIN-1 and EMILIN-2 affects the deposition and/or ECM organization of other proteins of the fibrillin microfibrillar system. Since we found that the formation of fibronectin fibers is required for proper deposition of EMILIN-1 and -2 we wanted to test whether EMILINs may also play a role in fibronectin matrix deposition and assembly. However, simultaneous depletion of EMILIN-1 and -2 did not have any impact on fibronectin fiber formation (Fig. 3A) excluding the possibility of interdependence of these proteins in the process of ECM fiber assembly by osteoblasts. Since it was reported that EMILINs modulate the activity of TGF-β-1, -2, and -3^5, 6^ we wanted to investigate whether EMILINs may be involved in targeting latent TGF-β binding proteins (LTBPs), the extracellular carriers of TGF-β, to the ECM. However, we found that expression and deposition of LTBPs such as LTBP-1 and LTBP-4 were not affected by EMILIN-1 and EMILIN-2 depletion in MC3T3-E1 cells (Fig. 3B–D). These findings suggest a temporal order of ECM organisation by osteoblasts whereby the fibronectin network is being formed prior to LTBP and EMILIN deposition.

**Figure 4:**
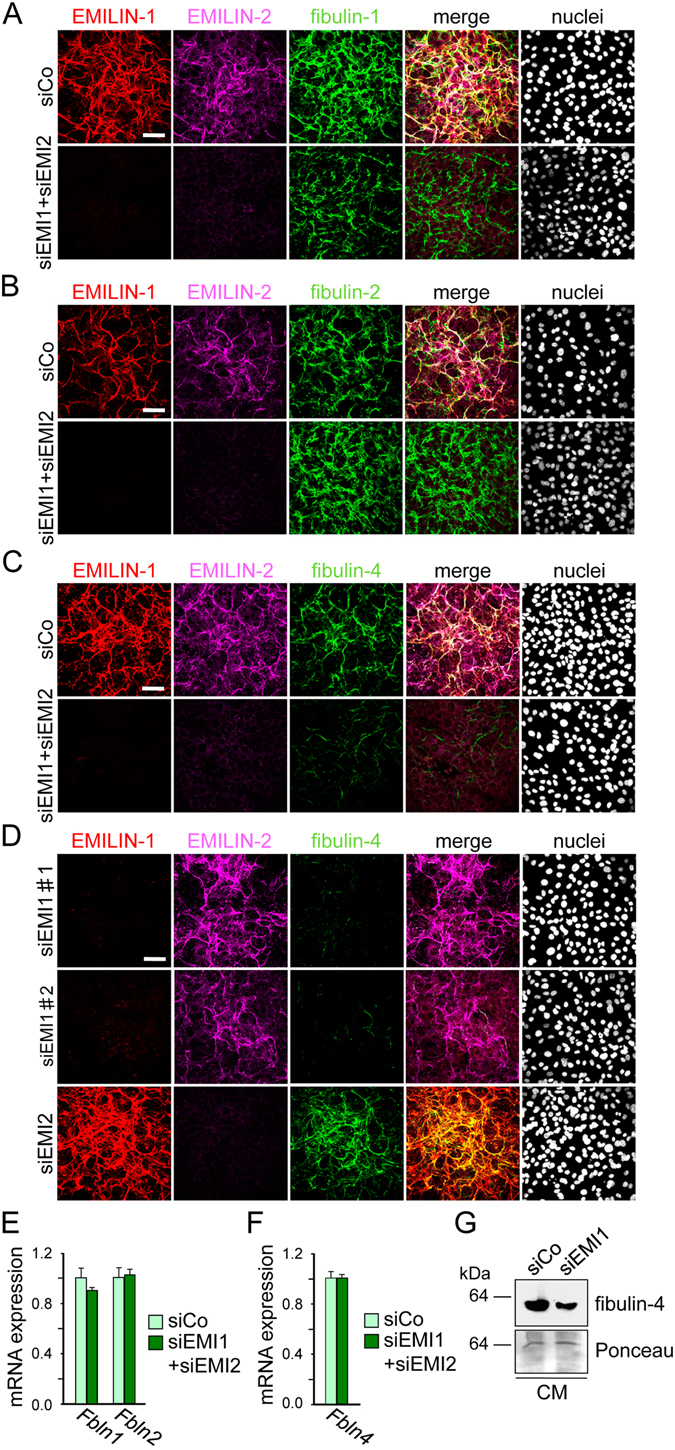
EMILIN-1 knockdown impairs the deposition of fibulin-4 in the ECM of MC3T3-E1 cells. (**A**,**B**) Expression and deposition of fibulin-1 and fibulin-2 in MC3T3-E1 cells after double knockdown for EMILIN-1 and EMILIN-2. MC3T3-E1 osteoblasts were reverse transfected with a mixture of siRNAs against EMILIN-1 (siEMI1) and EMILIN-2 (siEMI2) or with a control siRNA (siCo) and grown for 4 days. Confocal immunofluorescence microscopy of cells transfected with the different siRNAs and immunolabeled with antibodies against fibulin-1 (**A**) or fibulin-2 (**B**). Nuclei were stained with Hoechst. Scale bar, 75 μm. (**C**,**D**) Confocal immunofluorescence microscopy of cells for fibulin-4 after double knockdown of EMILIN-1 and EMILIN-2 (**C**), or single knockdown of EMILIN-1 or EMILIN-2 (**D**). Nuclei were stained with Hoechst. Scale bar, 75 μm. siEMI1#1 and siEMI1#2 represent two different siRNA sequences targeting EMILIN-1. (**E**) qRT-PCR analysis of *Fbln1*, *Fbln2* and *Fbln4* transcripts in cells transfected with the different siRNAs showed no significant differences (n = 3). **G**. Western blot analysis for fibulin-4 in conditioned medium (CM) of cells transfected with the different siRNAs. Protein loading is shown by Ponceau staining. All siRNA knockdown experiments were performed three times (n = 3).

Another important protein family associated to the fibrillin-microfibril/elastic fiber network is represented by fibulins^27^. In our *in vitro* model, EMILIN-1 and EMILIN-2 double knockdown did not grossly alter fibulin-1 or fibulin-2 expression and ECM deposition (Fig. 4A,B). In contrast to that, EMILIN-1 and EMILIN-2 double knockdown resulted in an almost complete loss of fibulin-4 in the ECM, whereas fibulin-4 transcript levels were comparable to control cells (Fig. 4C,E). To further investigate whether the loss of fibulin-4 extracellular deposition was linked to depletion of one of the two EMILIN proteins, we performed single knockdown experiments. While EMILIN-2 knockdown alone did not alter fibulin-4 deposition, single depletion of EMILIN-1, confirmed with two different siRNA sequences, was sufficient to prevent fibulin-4 deposition in the ECM even if the molecule was still secreted in the medium (Fig. 4D,F). This effect was even more visible when MC3T3-E1 cells were grown for longer culture times and harvested at 4, 7 and 10 days after transfection. While in control cells fibulin-4 fibers developed a thicker and abundant network over the 10 days of culture, in EMILIN-1 depleted cells fibulin-4 fiber formation was dramatically impaired (Supplementary Fig. S7B). To further confirm this finding in primary cells, we isolated primary calvarial osteoblasts from newborn wild-type mice and subjected them to the same siRNA treatments. As found for MC3T3-E1 cells, depletion of EMILIN-2 had no effect while EMILIN-1 knockdown impaired the deposition of fibulin-4 in the ECM of primary osteoblasts (Supplementary Fig. S8). To investigate whether fibulin-4 may also impact EMILIN-1 ECM incorporation, we performed siRNA mediated depletion of fibulin-4 in MC3T3-E1 cells. Our data show that fibulin-4 knock-down had no effect on EMILIN-1 fiber assembly but led only to a slight increase of EMILIN-1 transcript and protein levels (Supplementary Fig. S9). This shows that fibulin-4 is not required for EMILIN-1 matrix deposition by MC3T3-E1 cells. Despite the fact that fibulin-5 was previously reported to be present in the ECM of craniofacial bones of mice where its genetic ablation leads to dysregulated craniofacial skeletal development^28^, we found that it is not expressed by MC3T3-E1 cells, (Supplementary Fig. S7A).

### EMILIN-1 binds to fibulin-4, promotes fibulin-4 fiber formation, and co-localizes with fibulin-4 in calvarial bone

**Figure 5:**
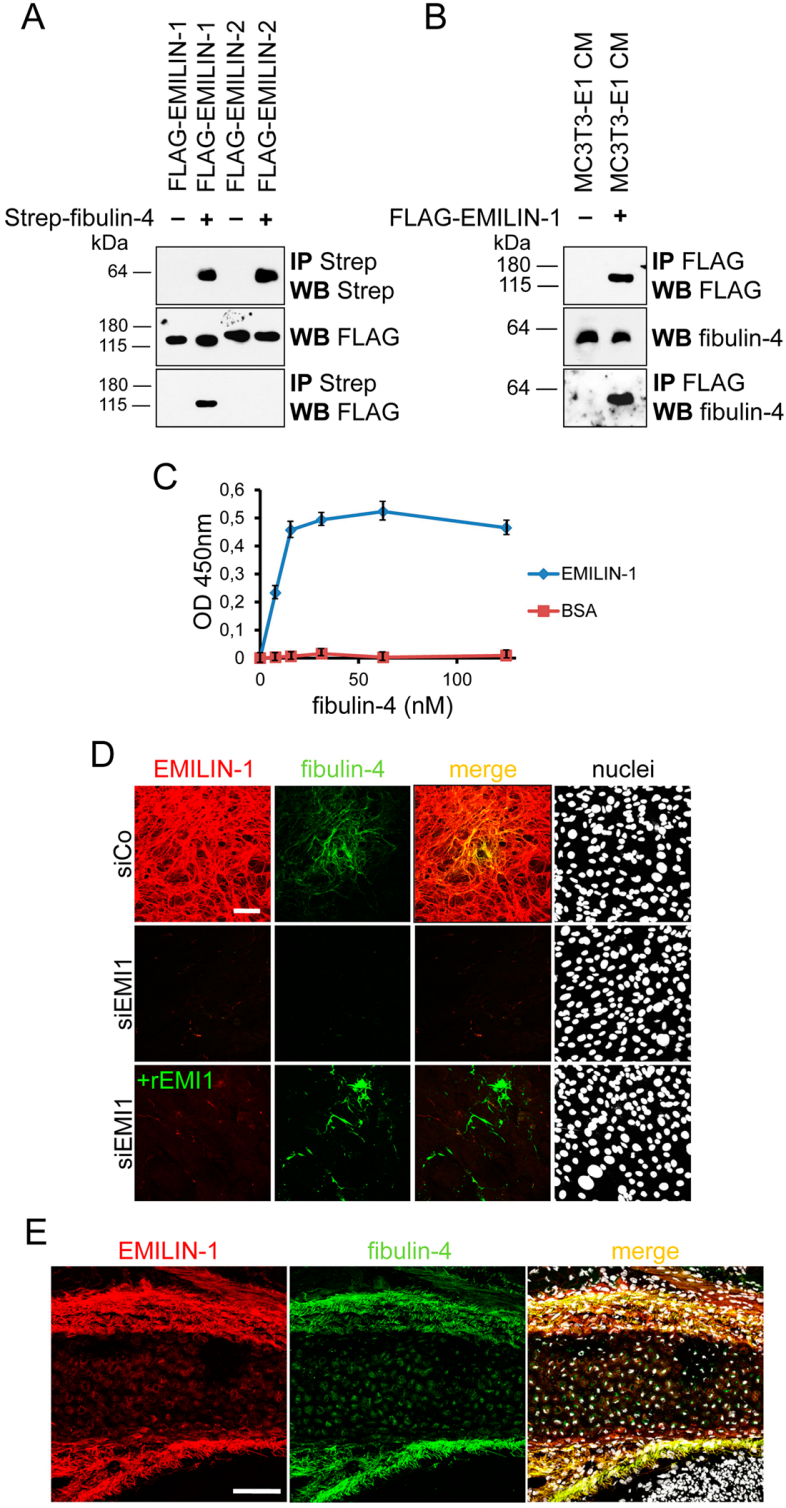
EMILIN-1 binds to fibulin-4, promotes fibulin-4 fiber formation in the ECM of MC3T3-E1 cells, and co-localizes with fibulin-4 in calvarial bone. (**A**) HEK293 EBNA cells were transfected with plasmids coding for murine EMILIN-1 and EMILIN-2 with a FLAG tag placed at their N-terminus. The obtained conditioned media were subjected to co-precipitation by incubation with recombinant 2 × strep-tagged murine fibulin-4. After washings, the elution fraction was analyzed by western blot with the indicated antibodies. (**B**) Conditioned media from control and FLAG-EMILIN-1 overexpressing EBNA cells were mixed with MC3T3-E1 conditioned medium. The mixed media were then subjected to immunoprecipitation with an anti-FLAG antibody and the resulting material probed by WB with a fibulin-4 specific antibody. (**C**) ELISA assay showing direct binding between coated recombinant human EMILIN-1 and soluble fibulin-4. (**D**) MC3T3-E1 osteoblasts were reverse transfected with a siRNA against EMILIN-1 (siEMI1) or with a control siRNA (siCo). Cells were grown on glass coverslip untreated or supplemented with 10 nM recombinant soluble EMILIN-1. After four days of culture cells were harvested, stained with indicated antibodies, and analyzed by confocal microscopy. Nuclei were stained with Hoechst. (**E**) Confocal immunofluorescence microscopy of EMILIN-1 and fibulin-4 on sagittal sections of newborn mouse skull shows co-localization of both proteins in calvarial bone. Scale bars, 75 μm.

To assess whether EMILIN-1 affects fibulin-4 matrix deposition through direct binding, we performed different *in vitro* binding assays. First, we found that EMILIN-1, but not EMILIN-2, is co-immunoprecipitated when recombinant fibulin-4 protein was added to the conditioned media of HEK293 EBNA cells transfected with plasmids coding for EMILIN-1 and EMILIN-2 with a FLAG tag (Fig. 5A). Next, by using conditioned media from cells over-expressing FLAG-tagged EMILIN-1 protein, we could immunoprecipitate endogenous fibulin-4 secreted in the medium of MC3T3-E1 cells (Fig. 5B). Direct binding was confirmed by ELISA assay using recombinant EMILIN-1 coated and fibulin-4 incubated at increasing concentrations in solution (Fig. 5C). Furthermore, we used recombinant EMILIN-1 protein to assess whether it was able to promote fibulin-4 deposition by MC3T3-E1 cells. Notably, addition of recombinant EMILIN-1 was able to restore fibulin-4 deposition in the ECM of EMILIN-1 depleted cells (Fig. 5D). Finally, by employing confocal immunofluorescence microscopy of EMILIN-1 and fibulin-4 on sagittal sections of newborn mouse skull we detected co-localization of both proteins in calvarial bone (Fig. 5E).

## Discussion

Despite the significant cellular functions EMILINs exert within the connective tissue space, little is known about where and how EMILINs are targeted and incorporated within the ECM architecture. This information is mandatory for a better understanding of how EMILINs work in concert with other molecular constituents of specialized tissue microenvironments to regulate tissue structure and function.

In this study, we provide for the first time more detailed information about the localization patterns of EMILINs within the ECM of cartilage and bone. So far EMILINs have been mostly studied in the context of cardiovascular diseases since genetic ablation of EMILIN-1 in mice results in structural alterations of elastic lamellae in the aortic wall and hypertension^5, 15^, while EMILIN-2 null mice show suppressed platelet aggregation and delayed clot retraction^18^. However previously, proteomic analysis of P3 and P21 cartilage extracts using label-free quantitative mass spectrometry revealed that EMILIN-1 protein is upregulated during postnatal cartilage maturation of mice as well as in a corresponding *in vitro* culture model^24^ suggesting that it may play a role in endochondral bone growth. Furthermore recently, a new EMILIN-1 mutation was identified in a patient with a history of skeletal and cartilage complications^22^ shedding new light on the significance of EMILINs in cartilage and bone. Clinical appearance of this patient, especially his personal history of aortic aneurysms was suggestive of MFS; however, he did not meet the revised diagnostic criteria for MFS^22^. This potential functional overlap between *EMILIN1* and *FBN1* in humans may be explained by the findings of our previous investigations where we showed that in skin and in culture of human dermal fibroblasts the ECM deposition of EMILIN-1 and -2 exclusively depends on the presence of the fibrillin microfibrillar network^20^. Fibrillin microfibrils are ubiquitous structures of the ECM composed of a scaffold of fibrillin-1 and fibrillin-2 (and fibrillin-3 in humans) “beads-on-a-string” like polymers with a diameter of 10–12 nm and a periodicity of 50–55 nm when imaged by electron microscopy^29, 30^. *In vivo*, fibrillin microfibrils are organized in supramolecular structures in which fibrillin polymers are associated to a variety of ligands with a tissue specific pattern. Among these proteins are elastin, MAGPs, versican, LTBPs, ADAMTSlike proteins, and fibulins^21^. Interestingly, in states of microfibrillar disease, such as in the murine GT-8 model of Marfan syndrome, characterized by progressive degradation of the fibrillin microfibril network^31^, we found that both EMILIN networks are affected^20^. Moreover, immunofluorescence of skin from the *EMILIN1* patient showed a similar punctate staining for EMILIN-1 at the dermal-epidermal junction as we detected in the GT-8 Marfan mouse model^20, 22^, suggesting that both EMILIN-1 and fibrillin-1 deficiency initiate similar pathogenetic mechanisms characterized by progressive tissue degradation.

However in this study, we found that, unlike in skin, fibrillin-1 fiber formation is not required for EMILIN-1 incorporation into the ECM of osteoblasts. Interestingly, in the same cultures fibrillin-1 is required for EMILIN-2 localization and also in cartilage tissues such as the annulus fibrosus fibrillin-2 and EMILIN-1 show specific co-localization (Fig. 1A). This is surprising since EMILINs showed only partial co-localization with fibrillin-2 in the skin^20^. Similar to our results with E14 mouse embryonic fibroblasts^20^ and NIH/3T3 cells, which were established from E17-19 mouse embryos^32^ (Supplementary Fig. 6), the deposition of EMILIN-1 by primary calvarial osteoblasts and MC3T3-E1 cells crucially depends on the formation of fibronectin. Similar findings were already reported for other proteins associated with fibrillin microfibrils. For instance, LTBP-1 incorporation into the ECM produced by osteoblasts and embryonic fibroblasts is controlled by fibronectin^33, 34^, while in primary dermal fibroblasts this process solely depends on fibrillin-1^35^. These differences we found between dermal fibroblasts, embryonic fibroblasts and osteoblasts point to cell-type specific and therefore context dependent matrix deposition requirements for each of the EMILINs reflecting differences in ECM architecture and function in the respective cellular microenvironment. Anchorage either to the fibrillin or the fibronectin network may impact EMILIN structure and presentation to the cell surface and thereby altering their functionality. Depending on the differences in ECM composition and architecture cellular microenvironments may promote or inhibit differentiation processes in response to the same extracellular stimuli such as TGF-β or Wnt ligands.

In this context, it is interesting that our data show EMILIN-1 localization to cartilage and bone, however, we detected discrepancies regarding the presence of EMILIN-2 and -3 in tissues and MC3T3-E1 cells. Previously, EMILIN-3 gene expression was suggested to take place at sites of mesenchymal condensations during cartilage and bone formation^36^. Further, *in situ* hybridization analysis of E14.5 mouse embryos showed EMILIN-3 mRNA expression in the perichondrium around sites of bone and cartilage formation in trunk, skull, and limbs^6^. However, while we also detected EMILIN-3 in calvarial bone of newborn mice it was not expressed and secreted by MC3T3-E1 cells (Supplementary Fig. 2) a preosteoblast cell line which has been established from newborn mouse calvaria and selected on the basis of high alkaline phosphatase (ALP) activity in the resting state^25^. This observed discrepancy suggests that EMILIN-3 is expressed and deposited before birth by embryonic mesenchymal cells. In MC3T3-E1 cells which represent postnatal preosteoblasts which have due to their selection for high ALP activity a strong tendency to differentiate into mature osteoblasts we found EMILIN-3 expression to be shut down. In contrast EMILIN-2 has not been reported to be present in cartilage or bone^12^. Consistent with that we did not detect EMILIN-2 in the ECM surrounding carvarial bone or mouse tracheal cartilage (Fig. 1B,C). However, we found that MC3T3-E1 cells are able to assemble ECM fibers positively stained for EMILIN-2. This finding allows to speculate that EMILIN-2 produced by MC3T3-E1 cells may serve as extracellular modulator of osteoblast maturation. EMILIN-2 directly interacts with Wnt1^8^, belonging to Wnt family members which were shown to be expressed in calvarial tissue and osteoblast cultures^37^. Since Wnt signaling is known to regulate osteoblastogenesis and therefore bone mass^38^, it may be plausible that EMILIN-2 produced by osteoblasts has a regulatory function in the maturation process of osteoblasts.

Here, we report that EMILIN-1 binds to and co-localizes with fibulin-4 in calvarial bone and that this interaction has, at least *in vitro*, functional consequences, as knockdown of EMILIN-1 in MC3T3-E1 and in primary calvarial osteoblasts is sufficient to impair the incorporation of fibulin-4 into the ECM. A similar effect, but involving fibulin-5, was reported in embryonic fibroblasts derived from EMILIN-1 knockout mice^14^. Despite these findings, mouse genetics showed that genetic ablation of EMILIN-1 does not fully recapitulate the most life threatening phenotypes of fibulin-4 and fibulin-5 knockout mice for instance in the aorta. Both EMILIN-1 and fibulin-5 null mice show defects in elastic fiber formation in the aorta^14, 39^ and show cardiovascular abnormalities such as high systolic blood pressure^5, 15^ or pulse pressures and compromised left ventricle diastolic function^40^, with stronger defects of elastogenesis induced by absence of fibulin-5^39^. However, knockout of fibulin-4 in mice leads to perinatal mortality due to an impairment of elastic fiber formation^41^. These data suggest that EMILIN-1 plays a role in the formation and maintenance of the elastic fiber system in the aorta but is not absolutely mandatory for the overall function of fibulin-4 or fibulin-5 in this microenvironment. This may be due to so far not investigated compensating mechanisms potentially involving other EMILINs or LTBPs. Since all EMILINs and also Multimerin-1, a closely related glycoprotein also carrying an EMI domain^4^, are expressed in postnatal aorta of mice^42^ it can be hypothesized that EMILINs function in a cooperative manner in the aortic wall and may be partly compensating for each other. Previously, similar cooperative mechanisms have been reported for fibulin-5 and fibulin-2 in the postnatal formation of the internal elastic lamina and in the maintenance of the adult vessel wall after injury^43^. Moreover, it is conceivable that upregulation of LTBP-4 may compensate for the lack of EMILIN-1 since it was shown that the long isoform of LTBP-4L is required for the linear deposition of fibulin-4 and its presence was shown to prolong survival^44, 45^. Overall, our findings in the ECM of osteoblast may be insightful for molecular mechanisms in the specialized microenvironments of blood vessels. EMILIN-1 may direct proper elastic fiber orientation to fulfill the required mechanical properties of blood vessels and may play a role in the development and progression of aortic tortuosity and aneurysm, both conditions initiated by fibulin-4 deficiency.

In this context, the discovery of a new and direct link between fibulin-4 and EMILIN-1 in the ECM produced by osteoblasts provides a better molecular understanding of pathogenic mechanisms underlying connective tissue disorders caused by mutations in fibulin-4 and EMILIN-1. Interestingly, the clinical presentation of a reported patient affected by a novel EMILIN-1 mutation shows overlapping features with those of patients carrying fibulin-4 or fibulin-5 mutations such as aortic aneurysms, joint laxity, and cutis laxa^22, 46^. One possible hypothesis for this clinical overlap may be that EMILIN-1 is required for proper ECM deposition of fibulin-4 which in turn modulates collagen homeostasis. Recently, it could be shown that smooth muscle cell-specific ablation of fibulin-4 in the aortic wall resulted in reduced collagen cross-linking and structural alterations of the collagen network indicated by poorly organized fibrils^47^. Fibulin-4 is known to bind lysyl oxidase (LOX), an elastin/collagen cross-linking enzyme essential for stabilization of collagen fibrils and for the integrity and elasticity of mature elastin^48^. Furthermore, it was reported that complete ablation of fibulin-4 in mice leads to unusually thick collagen fibrils and significantly reduced collagen cross-links in bone^49^. In addition, the amount of LOX in long bones and calvaria was strongly decreased and proteolytic activation of LOX was reduced in fibulin-4 deficient osteoblasts^49^. Therefore, it is reasonable to speculate that the recently reported human EMILIN-1 mutation which results in 50% reduction in secretion could result in a systemic connective tissue disorder at least in part by affecting fibulin-4 secretion and incorporation in the ECM with detrimental consequences for the collagen network. More studies will be required to address this hypothesis.

## Materials and Methods

### Ethics statement

This study was carried out in strict accordance with the German federal law on animal welfare, and the protocols were approved by the “Landesamt für Natur, Umwelt und Verbraucherschutz Nordrhein-Westfalen” for breeding (permit No. 84-02.04.2014.A397) and euthanasia (permit No. 84-02.05.40.14.115).

### Antibodies and recombinant proteins

A rat monoclonal antibody against mouse EMILIN-1 (clone 1007C11A8)^1^ was used. Rabbit and guinea pig affinity-purified antibodies for EMILIN-2 and EMILIN-3 were already described^6, 20^. Affinity purified rabbit polyclonal antibodies directed against mouse fibrillin-1 (pAb9543) and fibrillin-2 (868) were a kind gift from Dr. Lynn Sakai (Shriners Hospital for Children, Portland, OR, USA). Rabbit polyclonal antibody against fibronectin was from Sigma (St Louis, MO). Rabbit polyclonal anti LTBP-1 antibody was a kind gift from Dr. C. H. Heldin (Ludwig Institute for Cancer Research, Uppsala, Sweden). Polyclonal goat anti-LTBP-4 antibody was purchased from R&D Systems. Fibulin-1, -2, -4 and -5 antibodies were generated as previously described^50^. Recombinant murine C-terminally 2xStrepII tagged full length fibulin-4 and monomeric EMILIN-1 was expressed, and purified as previously described^44, 51^.

### Cell culture and siRNA transfections

MC3T3-E1 (subclone 4) and NIH/3T3 cells were purchased from ATCC and maintained in alpha Minimum Essential Medium supplemented with 10% FBS. For all the experiments, cells were grown in Dulbecco’s Modified Eagle’s medium (DMEM GlutaMAX, Invitrogen, Carlsbad, CA) supplemented with 10% FBS. For culturing NIH/3T3 cells in the presence of heparin, cells were changed into medium containing 100 µg/ml heparin (Sigma) one day after seeding. Primary calvarial osteoblasts were isolated from newborn mice as previously described^52^ and maintained in Dulbecco’s Modified Eagle’s medium (DMEM GlutaMAX, Invitrogen, Carlsbad, CA) supplemented with 10% fetal bovine serum and penicillin/streptomycin. For ECM network formation and RNA analysis, cells were seeded on uncoated glass coverslips or directly on plastic, respectively, at a density of 8 × 10^4^ cells/well in a 24-well plate. siRNAs transfections were carried out with Lipofectamine RNAiMAX (Invitrogen) according to the manufacturer’s instructions. Gene-specific siRNA and the AllStars Negative Control siRNA were purchased from Qiagen (Hilden, Germany). siRNAs used in this study are siEMI1#1: ACCCGAGGGACTGGAGAATAA; siEMI1#2: CACCGGCATGAGAAAGTGGAA; siEMI2#1: CAGGTTGCAGATGCAAAGCAA;

siEMI2#2: CAGGAGAGAGTTCCTGGAATA; siFbn1#1: ATGGTGCTTATTAAGACCAAA; siFbn1#2: AACGGAATGTGTATTAATGAA; siFbn2#1: CTCGACGAATGTCAAACCAAA;

siFbn2#2: CCCAGTCAACATGAAGTTCAA. Validated siRNAs from Thermo Fisher Scientific (Schwerte, Germany) were used to knockdown fibronectin (Fn; 165823, 165824) and fibulin-4 (Efemp2; 181952, 181953).

### Immunoelectron microscopy

Newborn mouse tail and trachea were labeled using *en bloc* diffusion of primary antibodies^20^ followed by secondary anti-rabbit, anti guinea pig, or anti-rat IgG conjugated with 5 or 10 nm gold particles.

### Immunofluorescence analysis

Mouse tissues were embedded in OCT Compound (Sakura, Alphen aan den Rijn, The Netherlands), and frozen in liquid nitrogen to generate sections of 7 μm. Sections or glass coverslips from cell culture were fixed at −20 °C in methanol/acetone, blocked in a PBS/1% bovine serum albumin solution, and subsequently incubated with primary and secondary antibodies diluted in the blocking solution. Sections were mounted using the Dako Fluorescence Mounting Medium (DAKO, Glostrup, Denmark) and visualized with a Leica SP5 confocal laser microscope. Pictures were processed with the ImageJ software.

### Real-time PCR

Cells were seeded in 24-well plates at 8 × 10^4^ cells/well and grown for 4 days. Total RNA was prepared by pipetting 1 ml Trizol^TM^ reagent (Invitrogen) into each well following the manufacturer’s protocol. RNA (0.5 μg per sample) was reverse transcribed using the Biorad iScript^TM^ cDNA synthesis kit (Bio-Rad, Hercules, CA). Triplicate samples were amplified using the SensiFAST SYBR Hi-ROX Kit (Bioline GmbH, Luckenwalde, Germany) in a StepOnePlus^TM^ Real-Time PCR Detection System (Applied Biosystems, Foster City, CA). Data analysis was performed using the 2^−ΔΔCt^ method and quantitated relative to the *Gapdh* gene. The primers used are provided in Supplementary Table S1.

#### *In vitro* binding assays

EBNA cells were transfected with expression plasmids coding for murine EMILIN-1 and EMILIN-2 cloned into pCS2 + vector with a FLAG-tag at their N-terminal positions or with constructs coding for EMILIN-1, -2, and -3 cloned with a HA-tag at their C-terminus or with an empty pCS2 + vector^6^. Subsequently cells were grown for 5 more days in serum free DMEM GlutaMAX medium (Invitrogen, Carlsbad, CA). The media were then collected and mixed with recombinant fibulin-4 protein gently shaking for 2 hours at 4 °C. The mixtures were then subjected to precipitation with Strep-Tactin Sepharose (IBA, Goettingen Germany). Precipitated material was washed three times with the washing solution (50 mM Tris-HCl, pH 7.5, 150 mM NaCl, 2 mM EDTA, 1% Triton X-100, and protease inhibitors) and proteins were resolved by SDS-PAGE and analyzed by western blotting. For the endogenous fibulin-4 binding assay, aliquots of FLAG-EMILIN-1 and empty vector conditioned media were subjected to immunoprecipitation with an anti-FLAG M2 affinity gel (Sigma) for 2 hours at 4 °C, washed three times and incubated overnight at 4 °C with conditioned serum free media generated from MC3T3-E1 cells. After three washings, proteins were resolved by SDS-PAGE and analyzed by western blotting. Used antibodies are M2 monoclonal anti-FLAG (Sigma) and monoclonal anti-HA-tag (clone HA-7) (Sigma).

#### Solid-phase binding assay

Multiwell plates were coated with purified human EMILIN-1 (100 nM/well) in PBS 1 M NaCl at 4 °C overnight. Coated wells were blocked with 5% nonfat dry milk in TBS at room temperature for 1 h. Recombinant fibulin-4 was serially diluted 1:2 in 2% milk, TBS and incubated in the wells for 2hs, followed by a 1 h incubation with monoclonal HRP-conjugated anti-Strep-tag antibody (IBA). Color reaction of the enzyme immunoassay was achieved using the TMB (3,3′,5,5′-tetramefhyl-benzidine) substrate Kit (Thermo Fisher Scientific, Waltham, MA, USA) and stopped with 0.1 M HCl. Absorbance was read at 450 nm using a Microplate Reader Sunrise (Tecan, Maennedorf, Switzerland).

#### Statistical analysis

Data are expressed as mean ± standard deviation (SD). The significance of differences between groups was determined by an unpaired Student’s t test. Values of P ≤ 0.05 were considered significant.

## Electronic supplementary material

Supplementary Information
