# Supplementary material for: Fibulin-4 deposition requires EMILIN-1 in the extracellular matrix of osteoblasts
Source: Sci Rep. 2017 Jul 17;7:5526. doi: 10.1038/s41598-017-05835-7 (PMC5514116; doi:10.1038/s41598-017-05835-7)
Supplement: Supplementary file 1 — Supplementary Information [file 41598_2017_5835_MOESM1_ESM.pdf]

Supplementary information

Fibulin-4 deposition requires EMILIN-1 in the extracellular matrix of osteoblasts

Alvise Schiavinato[1], Douglas R. Keene[2], Thomas Imhof[1,3], Roberto Doliana[4], Takako Sasaki[5], and Gerhard Sengle[1,6]*

[1]Center for Biochemistry, Medical Faculty, University of Cologne, Cologne, Germany

[2]Shriners Hospital for Children, Portland, Oregon, USA

[3]Institute for Dental Research and Oral Musculoskeletal Biology, Medical Faculty, University of Cologne, 50931 Cologne, Germany

[4]Experimental Oncology 2, CRO, IRCCS, National Cancer Institute, Aviano, PN, Italy

[5]Department of Biochemistry II, Faculty of Medicine, Oita University, Oita 879-5593, Japan

[6]Center for Molecular Medicine Cologne (CMMC), University of Cologne, Cologne, Germany

*Author for correspondence:

Gerhard Sengle, Center for Biochemistry, Medical Faculty, University of Cologne, Joseph-Stelzmann- Street 52, D-50931 Cologne, Germany, phone: +49 (0)221 47897260, fax: +49 (0)2214786977, email: gsengle@uni-koeln.de

Supplementary Table S1. Sequences of qPCR primers used.

Gene	Primer name	Sequence
Gapdh	Gapdh FOR<br>Gapdh REV	CCACCCAGAAGACTGTGGAT<br>TTCAGCTCTGGGATGACCTT
Fbn1	fbn1 FOR<br>fbn1 REV	GATCAACGGCTACCCAAAAC<br>GTTGGCTTCCATCTCAGACC
Fbn2	fbn2 FOR<br>fbn2 REV	TAAGAATGGCCGATGTGTG<br>TTCAAGCACATGTTGGTCGT
Emilin1	Emi1 FOR<br>Emi1 REV	TGTGCCTACGTGGTGACTC<br>CGGTACATGATACTTCGGGAAC
Emilin2	Emi2 FOR<br>Emi2 REV	TCCATCCGCCCTGGTGTAT<br>GCAGTCTGGTCCTCTAAAGCC
Fbln1	Fbln1 FOR<br>Fbln1 REV	CCGCCAAGAGAAAACAGACAC<br>CGGGTGAACTCTCGAAAGGTG
Fbln2	Fbln2 FOR<br>Fbln2 REV	CATGCTCTCCTGCTGTGAAG<br>GCCATCTCCATCTCTGAAAC
Fbln4	Fbln4 FOR<br>Fbln4 REV	GAGCAGCCTTCATCCATTGT<br>AACGGATCTGAAAGGCATTG
Ltbp1	Ltbp1 FOR<br>Ltbp1 REV	TGCGATTGCTTTGATGGATA<br>GCTCGCTGCATTCATTTACA
Ltbp4	Ltbp4 FOR<br>Ltbp4 REV	GTCTCCAACGAGAGCCAGAG<br>ACGAGATCAGGTCCCACTTC

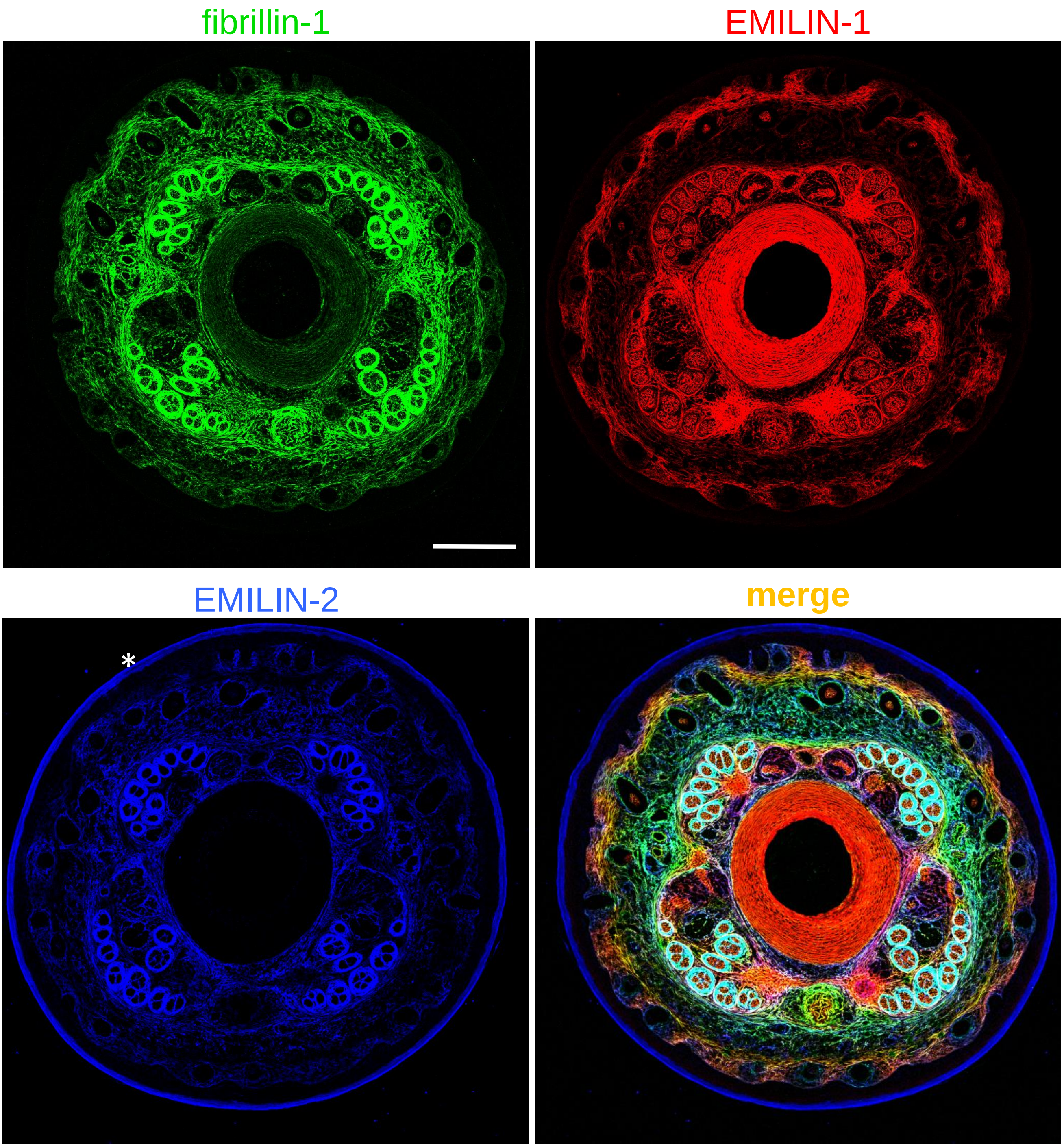

Supplementary Figure S1. Distribution of fibrillin-1, EMILIN-1 and EMILIN-2 in newborn mouse tail. Confocal immunofluorescence microscopy showing localization of fibrillin-1, EMILIN-1 and -2 in transverse sections of newborn mouse tail. The asterisk marks non-specific staining of the epidermis when anti-EMILIN-2 antibody was used (lower panel). Scale bar: 250 μm.

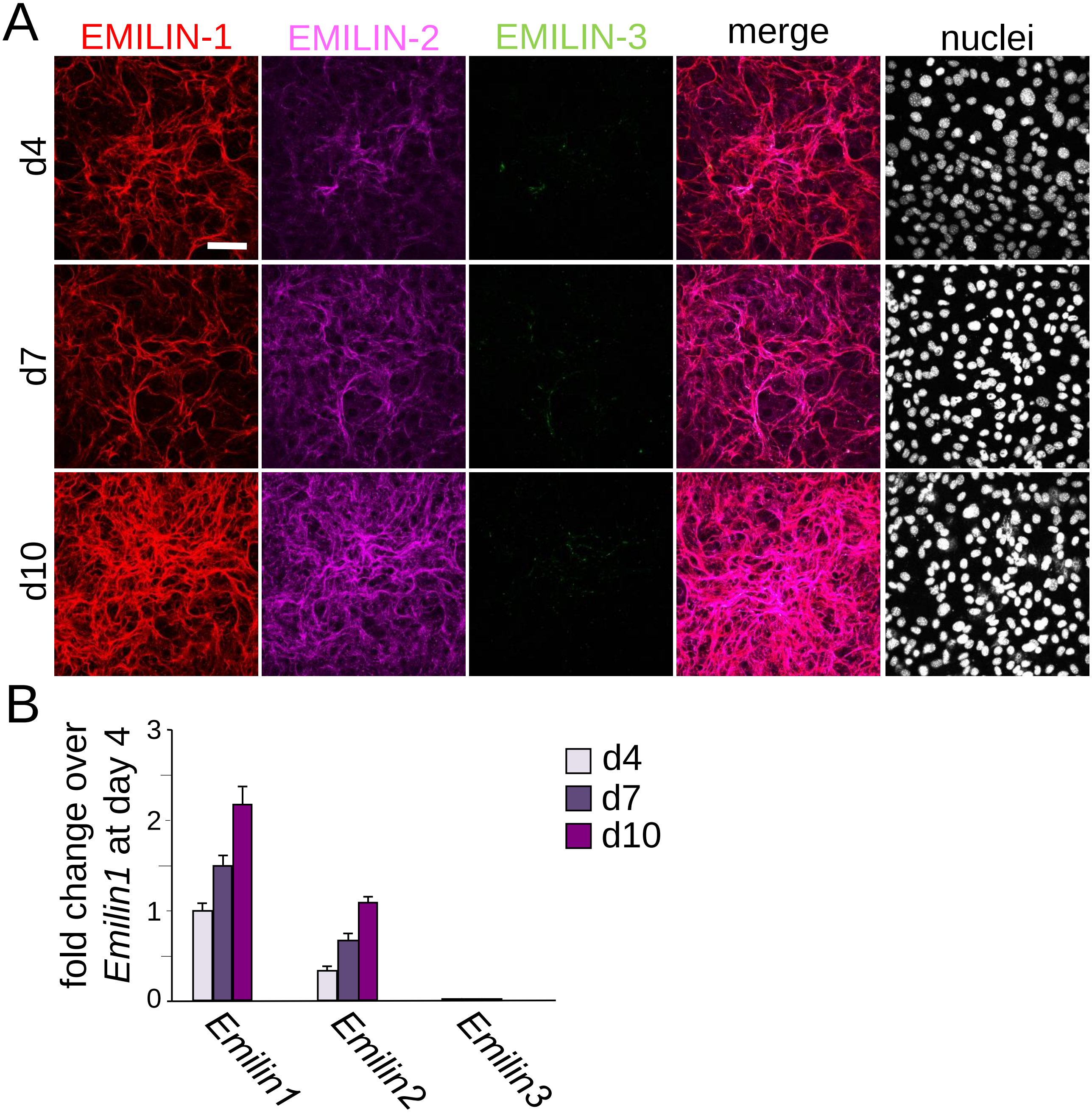

Supplementary Figure S2. Deposition of EMILINs in extracellular matrix of MC3T3-E1 cells.

A. Immunofluorescence analysis of MC3T3-E1 cells grown for the indicated culture times (d4-d10) and labelled with antibodies against the three different EMILINs. Nuclei were stained with Hoechst. Note the absence of EMILIN-3 from the extracellular matrix of these cells at all analyzed culture times. Scale bar, 75 µm. **B.** mRNA Expression of EMILINs in MC3T3-E1 cells. Cells were grown on plastic and harvested after 4 days (d4), 7 days (d7) or 10 days (d10) of culture. After RNA extraction, expression levels were measured by qPCR analysis. Expression level of *Emilin1* at day 4 was set as 1.

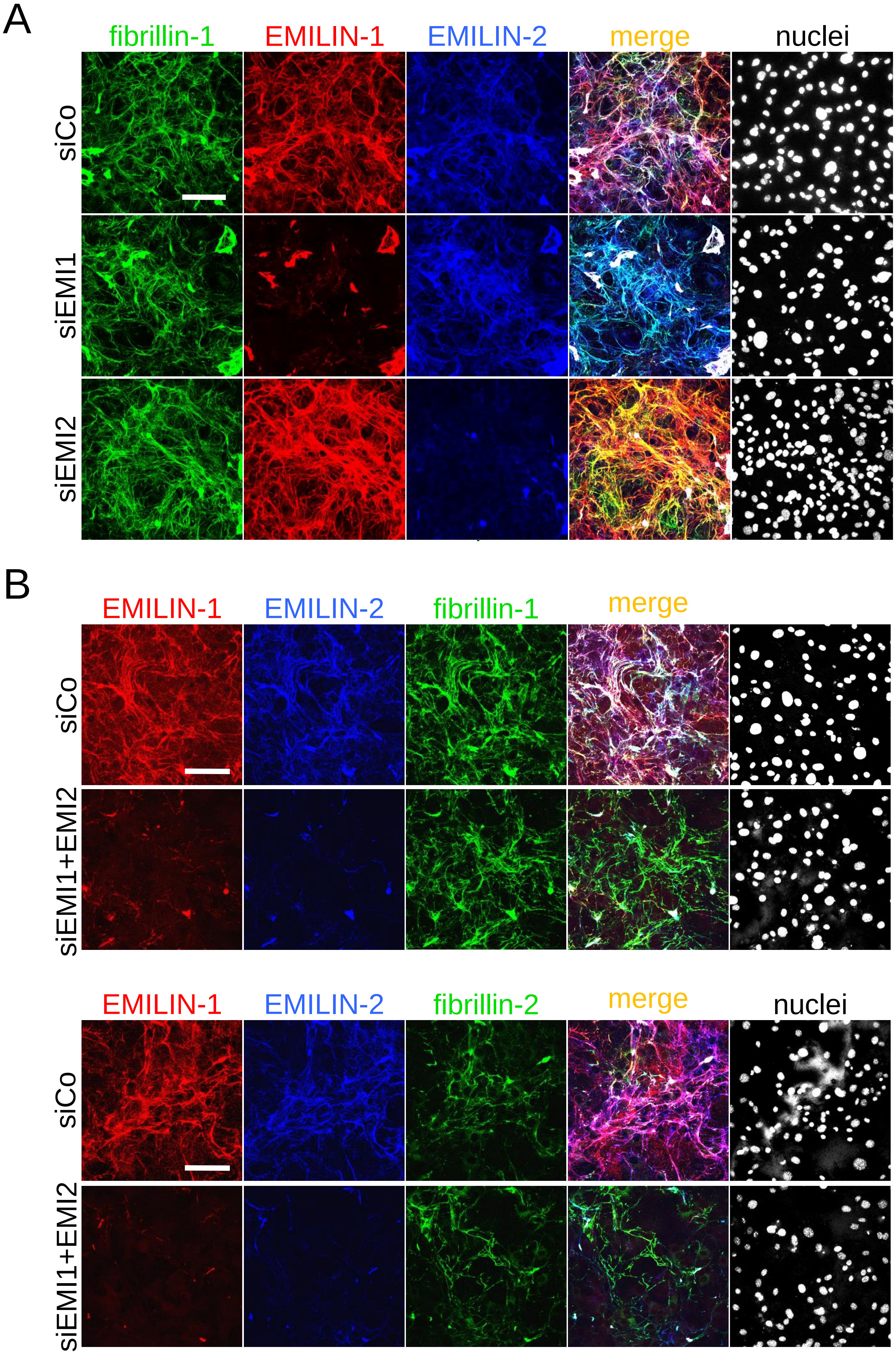

Supplementary Figure S3. EMILIN-1 and EMILIN-2 are not depending on each other for their assemby and are not required for fibrillin fibrillogenesis in primary calvarial osteoblasts.

A. Primary calvarial osteoblasts were reverse transfected with the indicated EMILIN siRNAs, plated on glass coverslips and grown for four days. The cultures were then stained with the indicated antibodies. As previously shown for dermal fibroblasts, single EMILIN knockdown had no effect on the assembly of the other EMILIN and on the incorporation of fibrillin-1 into the extracellular matrix. (**B**) After simultaneous EMILIN-1 and EMILIN2 knockdown, fibrillin-1 and fibrillin-2 network formation was investigated by immunostaining on transfected cells cultured for four days. As demonstrated for dermal fibroblasts, EMILINs were found not to be essential for fibrillin fibrillogenesis. Scale bars: 75 μm.

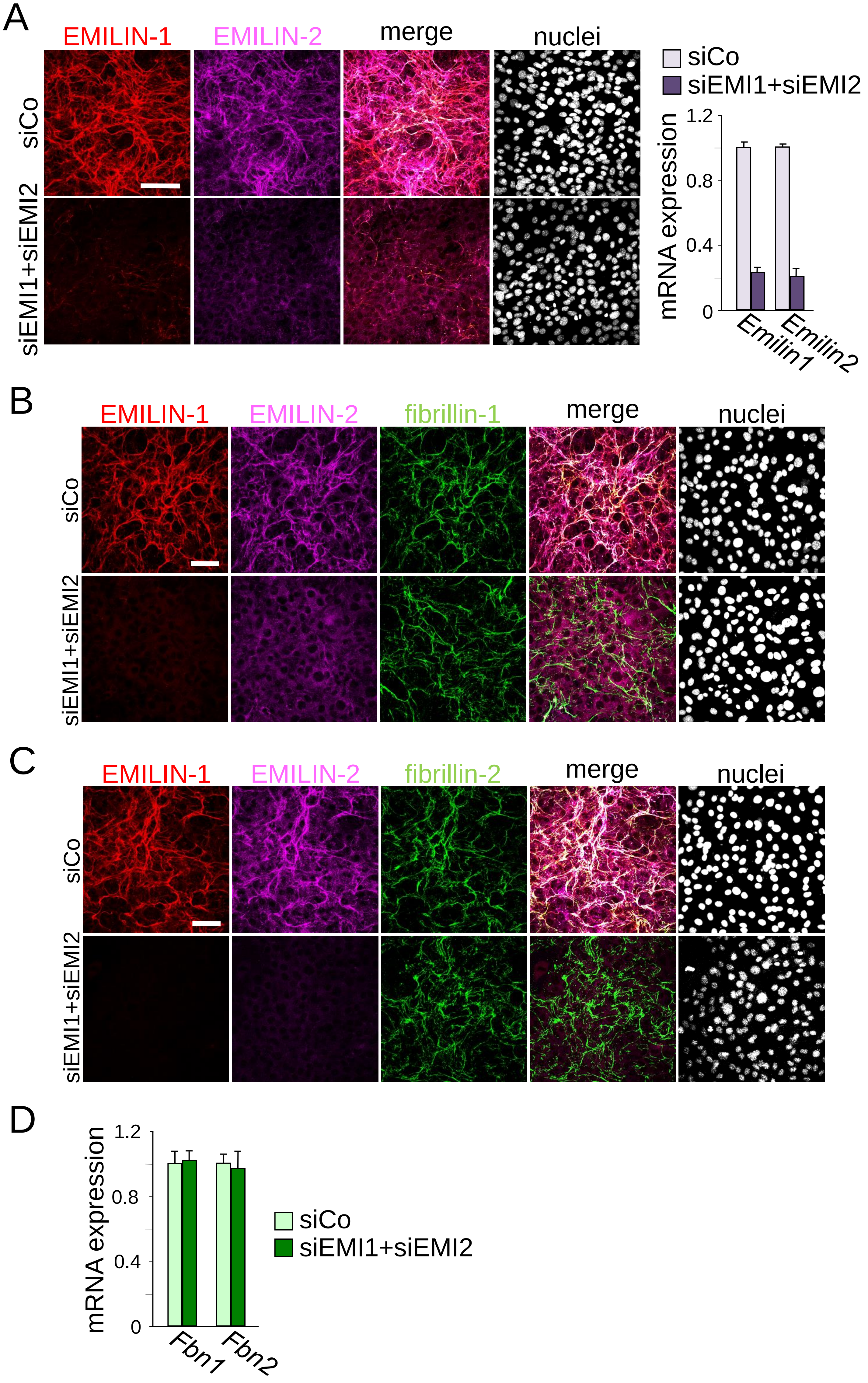

Supplementary Figure S4. Expression and matrix deposition of fibrillins in MC3T3-E1 cells after simultaneous depletion of EMILIN-1 and EMILIN-2. A. siRNA mediated depletion of EMILIN-1 and -2 in MC3T3-E1 cells. Cells were reverse transfected with a mixture of siRNAs specific for EMILIN-1 and EMILIN-2 (siEMI1+siEMI2) or with a control siRNA (siCo). After 4 days, cells were harvested and analyzed by immunofluorescence using the indicated antibodies ,and qPCR was conducted to monitor *Emilin1* and *Emilin2* messenger RNA levels (n=3). Scale bar, 75 µm. **B, C.** MC3T3-E1 osteoblasts were transfected with a mixture of siRNAs against *Emilin1* (siEMI1) and *Emilin2* (siEMI2) or with a control siRNA (siCo) and grown for 4 days. Confocal microscopy of cells transfected with the different siRNAs after immunolabeling with antibodies against fibrillin-1 (**B**) or fibrillin-2 (**C**). Nuclei were stained with Hoechst. Scale bar, 75 µm. **D.** qPCR analysis of fibrillin-1 and fibrillin-2 transcript levels in cells transfected with the different Emilin siRNAs (n=3) showed no significant differences.

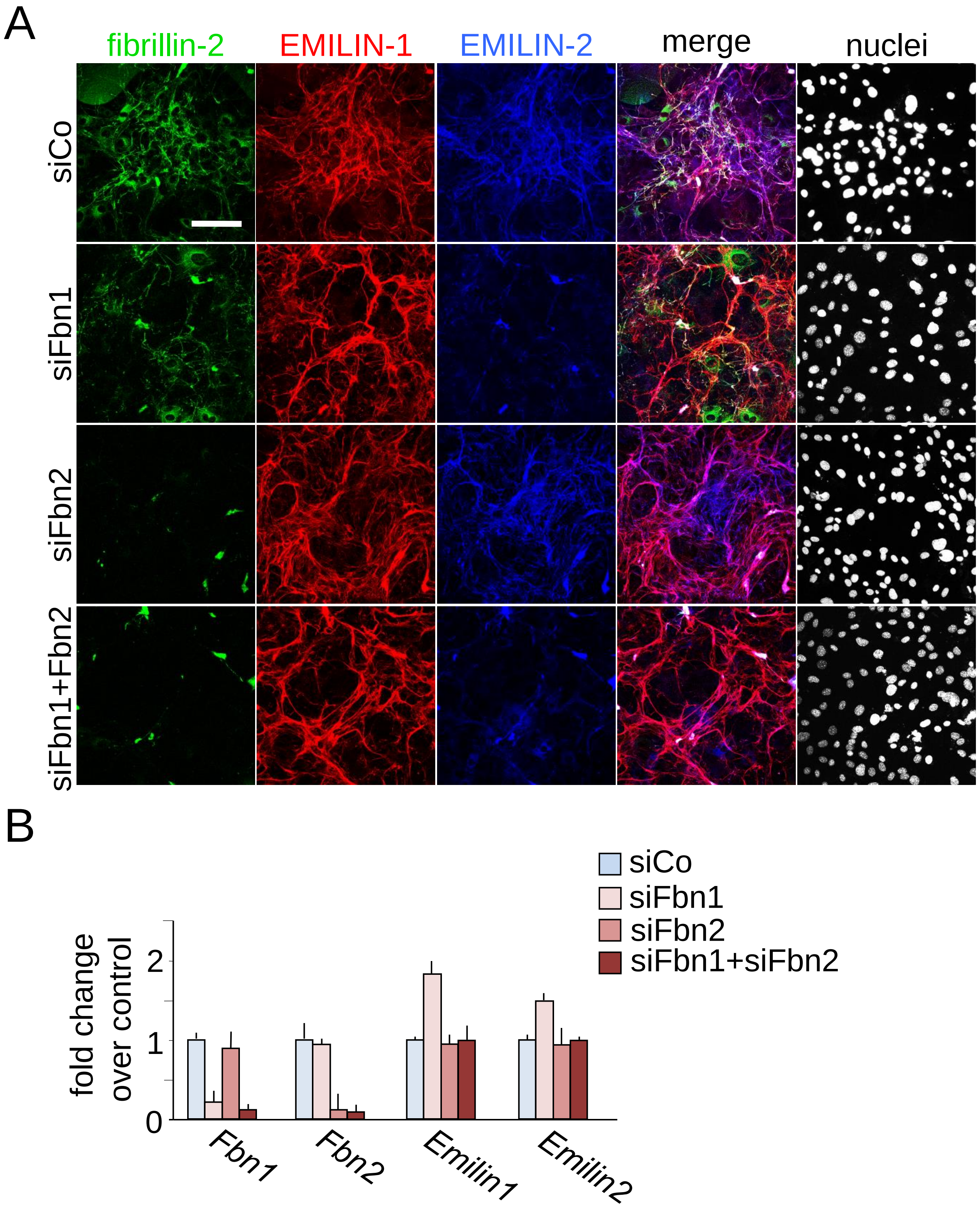

Supplementary Figure S5. Depletion of both fibrillins does not affect incorporation of EMILIN-1 into the matrix of primary calvarial osteoblasts. A. Confocal immunofluorescence microscopy of reverse transfected primary calvarial osteoblasts stained with the indicated antibodies to show the effect of siRNA treatment on fibrillin-2, EMILIN-1, and EMILIN-2 incorporation. Knockdown of fibrillin-1 alone was sufficient to impair EMILIN-2 incorporation into the extracellular matrix, while even the double knockdown of fibrillin-1 and fibrillin-2 did not affect EMILIN-1 fiber assembly. Scale bars: 75 µm. **B.** siRNA mediated knockdown of fibrillins does not significantly impact transcript levels of EMILINs (n=3).

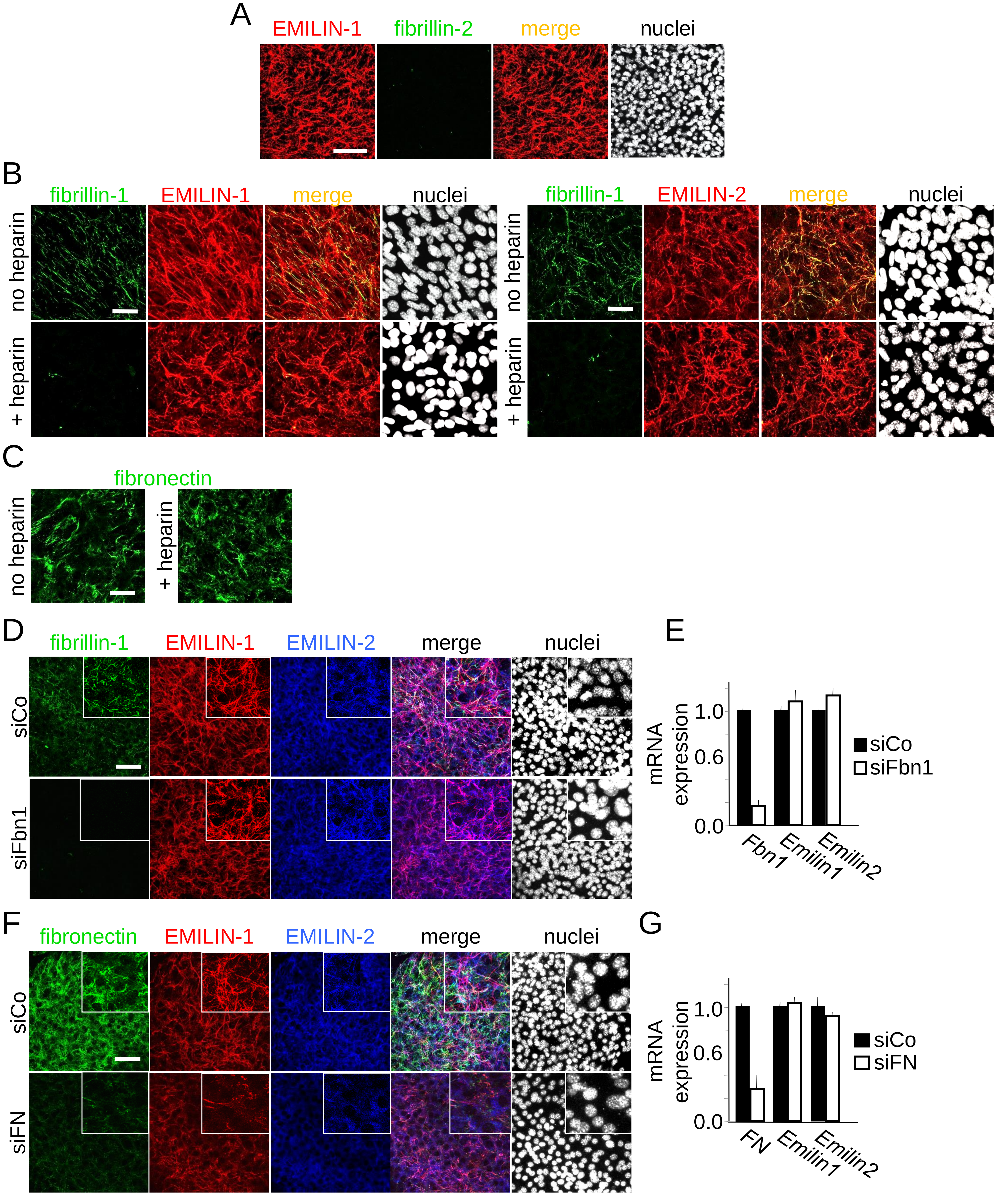

Supplementary Figure S6. EMILIN-1 and EMILIN-2 network formation by NIH/3T3 embryonic fibroblasts depends on fibronectin. **A**. Confocal immunofluorescence microscopy of NIH/3T3 cells plated on glass coverslips and grown for four days for extracellular matrix production. Fixed cells were then costained with antibodies for EMILIN-1 and fibrillin-2. Fibrillin-2 was found to be absent from the extracellular matrix of these cells. **B**. Confocal immunofluorescence microscopy of NIH/3T3 cells after heparin treatment which inhibits fibrillin-1 fiber formation. NIH/3T3 cells were plated on glass coverslips and the day after seeding the cells were changed into medium containing 100 µg/ml heparin or no heparin. Cells were fixed after four days and stained with the indicated antibodies. Heparin treatment inhibited fibrillin-1 fibrillogenesis but did not block EMILIN-1 and EMILIN-2 assembly. **C**. Culturing in presence of soluble heparin was also not affecting fibronectin deposition. **D.** siRNA mediated knockdown of fibrillin-1 had no effect on EMILIN-1 and EMILIN-2 distribution. (**B**) Real-time PCR revealed no changes in EMILIN-1 or EMILIN-2 mRNA expression after fibrillin-1 knockdown (*n=3*). **F.** Fibronectin is required for EMILIN-1 and EMILIN-2 assembly by NIH/3T3 embryonic fibroblasts. NIH/3T3 fibroblasts were reverse transfected with control (siCo) or fibrillin-1 siRNAs, cultured for four days and analyzed with confocal immunofluorescence microscopy for network formation utilizing the indicated antibodies **G**. siRNA mediated depletion of fibronectin did not reduce EMILIN-1 or EMILIN-2 mRNA expression (*n=3*). All scale bars: 50 µm. Boxed areas represent a twofold magnification.

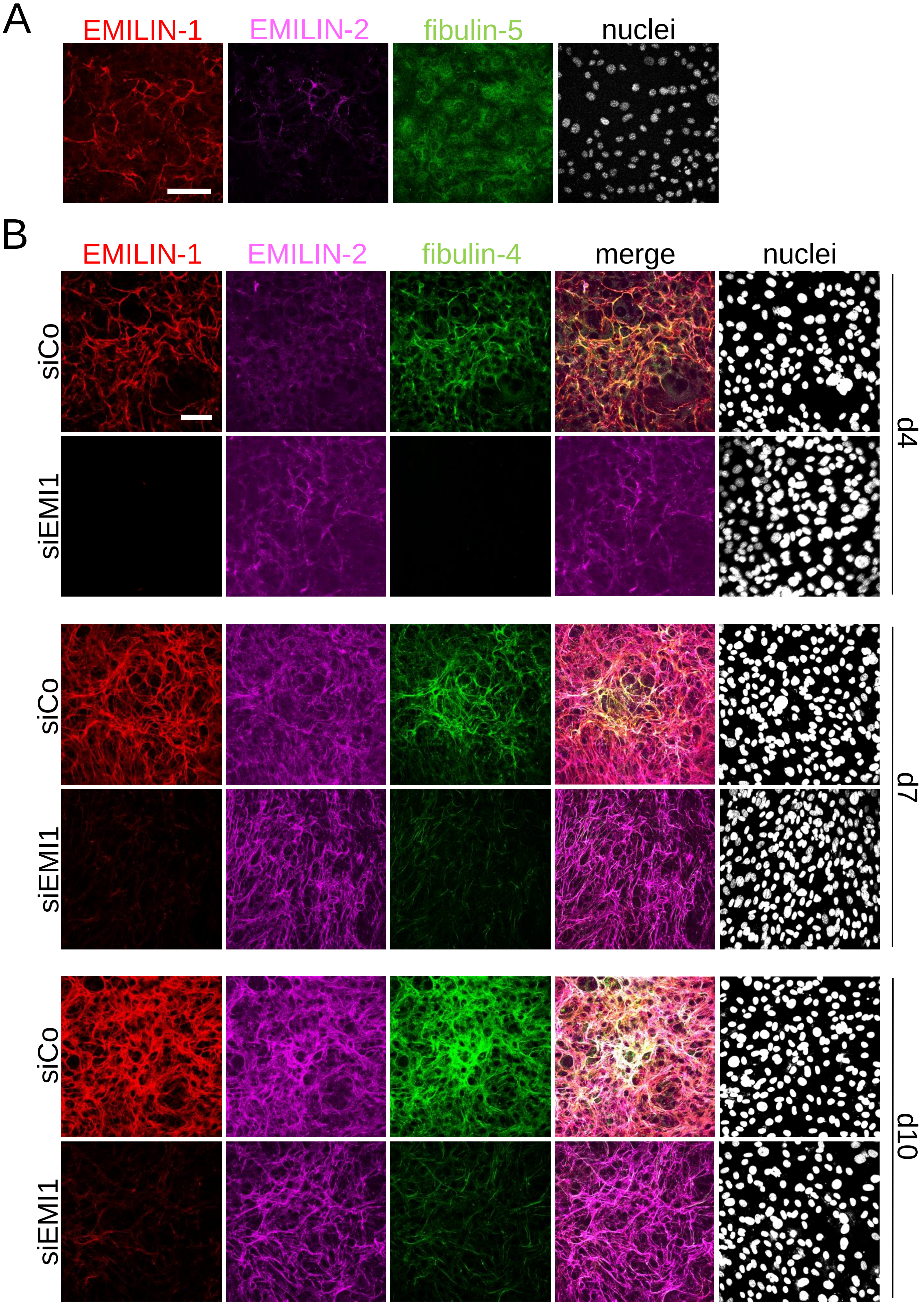

Supplementary Figure S7. Confocal immunofluorescence microscopy analysis of extracellular fibulin-5 and time course analysis of fibulin-4 ECM deposition when EMILIN-1 is depleted in MC3T3-E1 cells. A. MC3T3-E1 cells do not deposit fibulin-5 in culture. Cells were grown on glass coverslips and stained with the indicated antibodies. Note the absence of fibulin-5 positive extracellular fibers. Scale bar, 75 µm. **B.** Time course analysis of fibulin-4 ECM deposition when EMILIN-1 is depleted in MC3T3-E1 cells. MC3T3-E1 osteoblasts were reverse transfected with a siRNA against EMILIN-1 (siEMI1) or with a control siRNA (siCo) and grown for 4 days (d4), 7 days (d7) or 10 days (d10) before harvesting. Immunofluorescence for fibulin-4 was subsequently performed and cells were analyzed by confocal microscopy. Nuclei were stained with Hoechst. Scale bar, 75 µm.

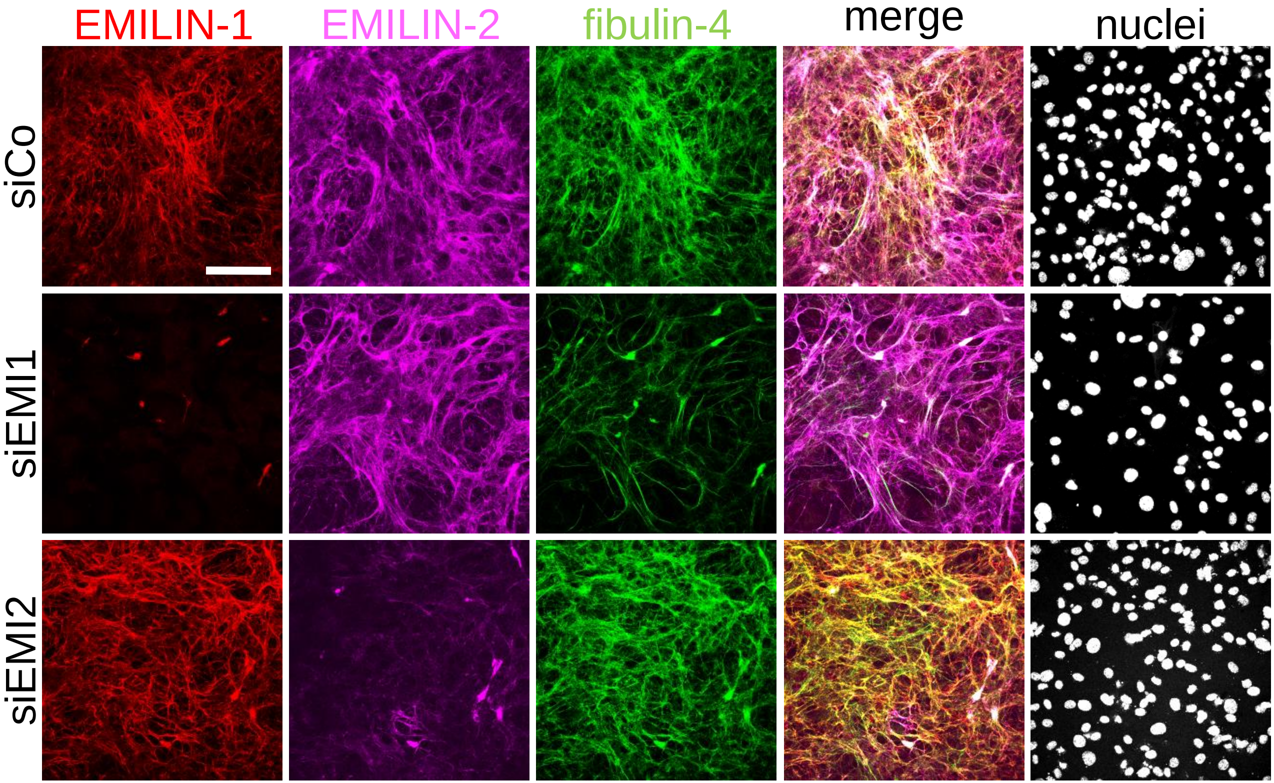

Supplementary Figure S8. siRNA mediated EMILIN-1 knockdown in primary calvarial osteoblasts impacts fibulin-4 ECM deposition. Primary osteoblasts isolated from newborn mouse calvaria were reverse transfected with siRNAs specific for *Emilin1* (siEMI1) or *Emilin2* (siEMI2) or with a control siRNA (siCo). After 4 days of culture on glass coverslips, cells were harvested and stained with the indicated antibodies. A reduction of fibulin-4 fiber network formation was only observed for cells treated with siEMI1. Scale bar, 75 µm.

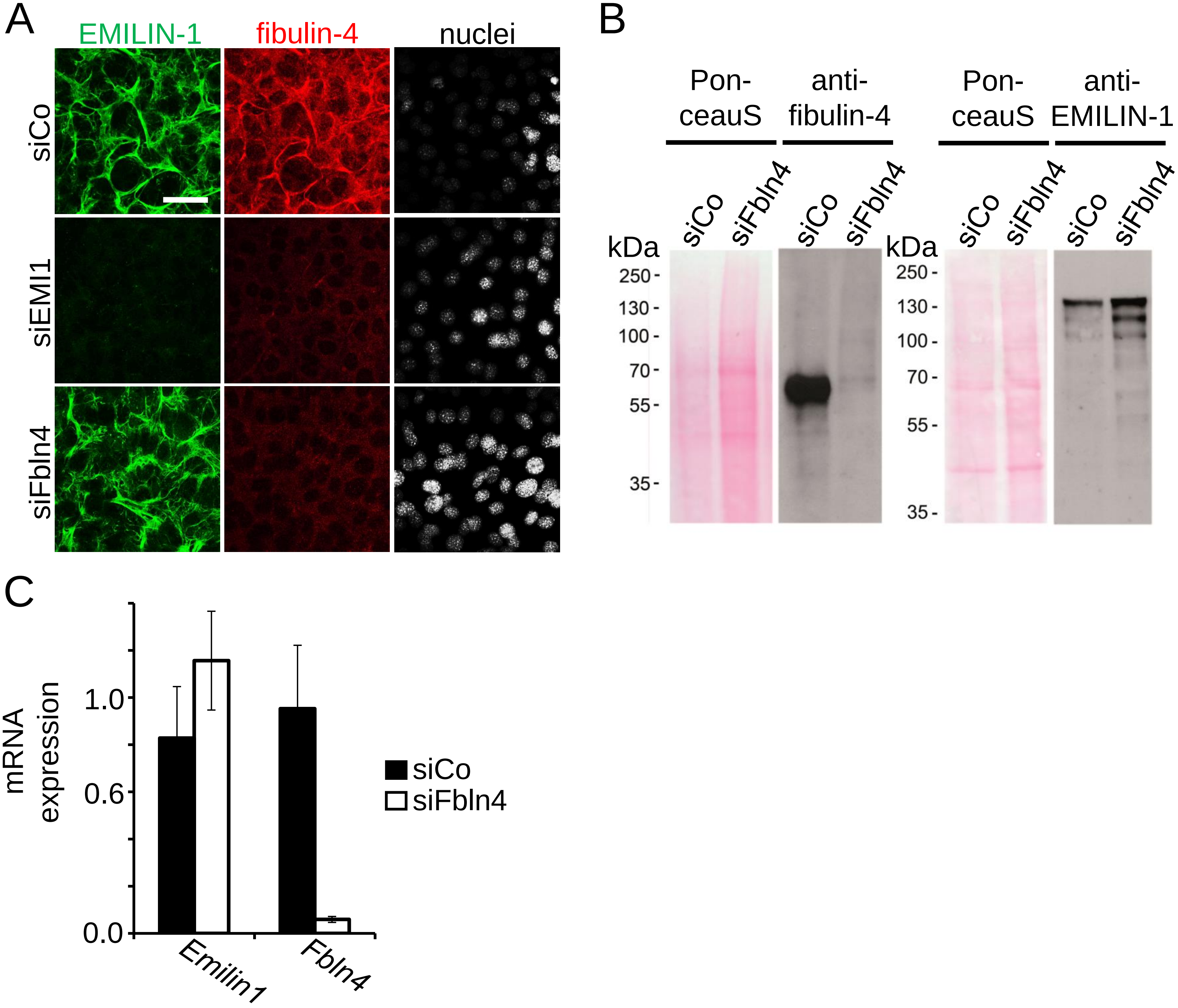

Supplementary Figure S9. siRNA mediated fibulin-4 knockdown in MC3T3-E1 calvarial osteoblasts does not impact EMILIN-1 ECM deposition or expression. A. Confocal immunofluorescence microscopy of MC3T3-E1 after siRNA mediated depletion of fibulin-4 or EMILIN-1. While knockdown of EMILIN-1 prevented fibulin-4 fiber asslembly EMILIN-1 fiber formation was not influenced by fibulin-4 siRNA depletion. **B.** Western blot analysis of cell culture supernatants showed complete inhibition of fibulin-4 secretion after knockdown, however, EMILIN-1 levels were not affected. **C.** qPCR showed effective knockdown of fibulin-4 transcripts, however, Emilin1 mRNA expression was not perturbed (n=3). Scale bar, 100 µm.
